# Supplementary material for: Imagined speech can be decoded from low- and cross-frequency intracranial EEG features
Source: Nat Commun. 2022 Jan 10;13:48. doi: 10.1038/s41467-021-27725-3 (PMC8748882; doi:10.1038/s41467-021-27725-3)
Supplement: Supplementary file 1 — Supplementary Information [file 41467_2021_27725_MOESM1_ESM.pdf]

Imagined speech can be decoded from low- and cross-frequency intracranial EEG features

Supplementary Information

Timothée Proix, Jaime Delgado, Andy Christen, Stephanie Martin,

Xing Tian, David Poeppel, Werner K. Doyle, Orrin Devinsky,

Luc Arnal, Pierre Mégevand, Anne-Lise Giraud

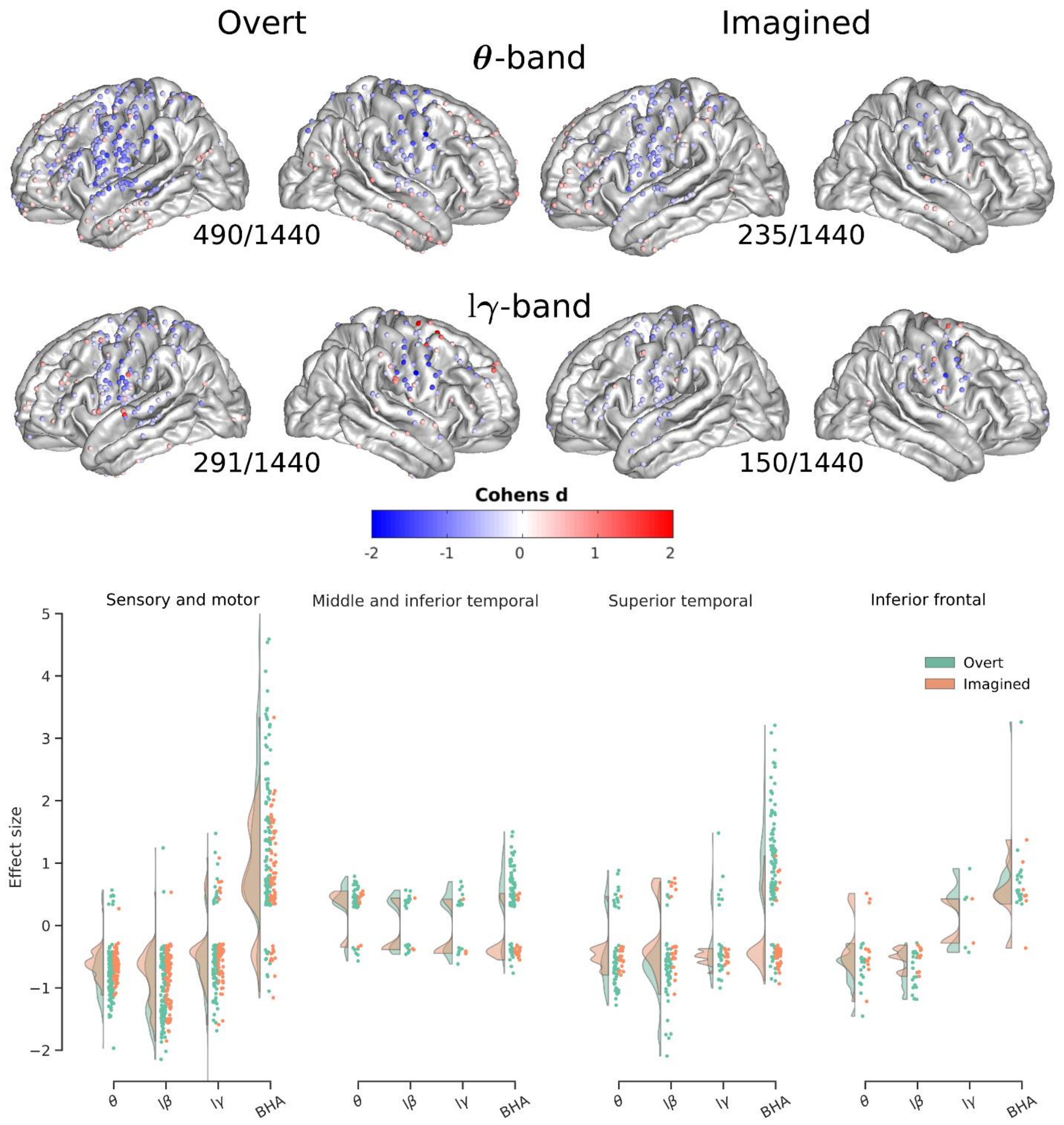

Supplementary Figure 1: Spatial organization of power spectrum deviations from baseline elicited by overt and imagined speech. Top: effect sizes (Cohen's d) for significant cortical sites across all participants and studies during overt and imagined speech compared to baseline (only significant electrodes are shown, t-tests, FDR-corrected, target threshold α = 0.05). The number of significant electrodes over the total number of electrodes is indicated below each plot. Left column: overt speech. Right column: imagined speech. Results are pulled across all studies, results for separated studies as shown in Supp. Fig 2-4. Bottom: effect sizes for each ROI and frequency band. Source data are provided as a Source Data file.

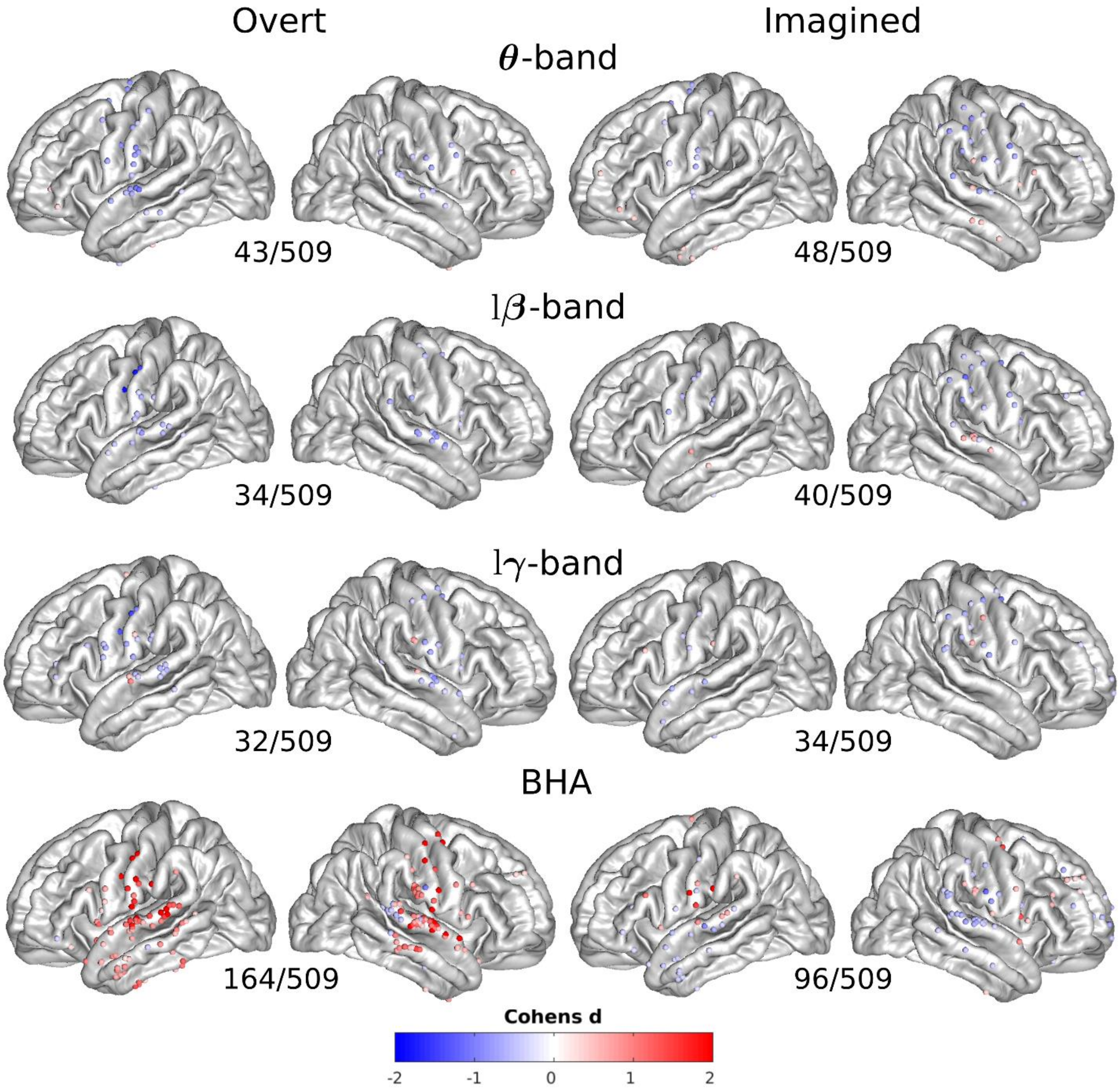

Supplementary Figure 2: Spatial organization of power spectrum deviations from baseline elicited by overt and imagined speech for study 1. Effect sizes (Cohen's d) for significant cortical sites across participants of study 1 during overt and imagined speech compared to baseline (only significant electrodes are shown, t-tests, FDR-corrected, target threshold $\alpha = 0.05$). The number of significant electrodes over the total number of electrodes is indicated below each plot. Left column: overt speech. Right column: imagined speech.

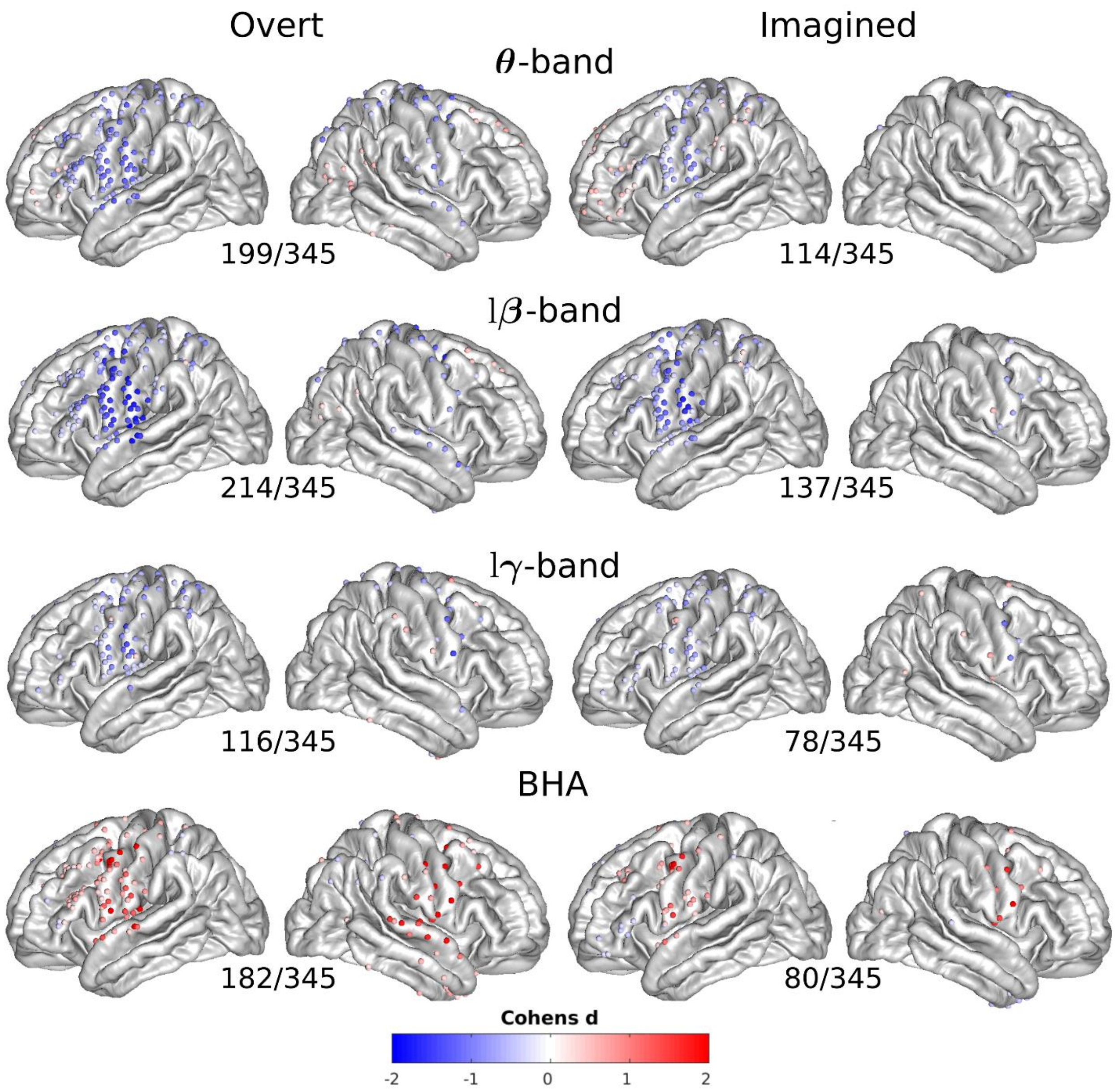

Supplementary Figure 3: Spatial organization of power spectrum deviations from baseline elicited by overt and imagined speech for study 2. Effect sizes (Cohen's d) for significant cortical sites across participants of study 2 during overt and imagined speech compared to baseline (only significant electrodes are shown, t-tests, FDR-corrected, target threshold α = 0.05). The number of significant electrodes over the total number of electrodes is indicated below each plot. Left column: overt speech. Right column: imagined speech.

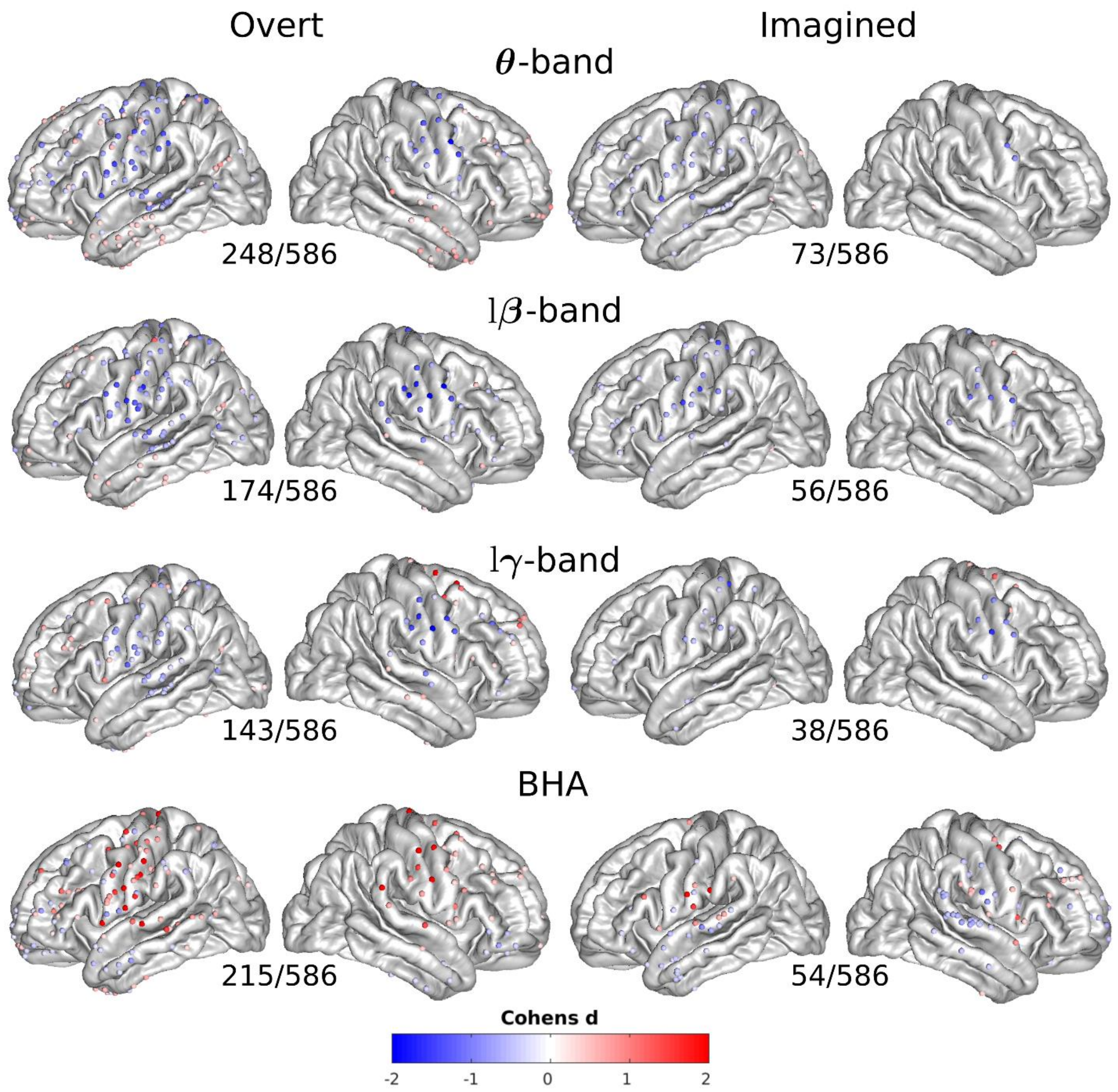

Supplementary Figure 4: Spatial organization of power spectrum deviations from baseline elicited by overt and imagined speech for study 3. Effect sizes (Cohen's d) for significant cortical sites across participants of study 3 during overt and imagined speech compared to baseline (only significant electrodes are shown, t-tests, FDR-corrected, target threshold $\alpha = 0.05$). The number of significant electrodes over the total number of electrodes is indicated below each plot. Left column: overt speech. Right column: imagined speech.

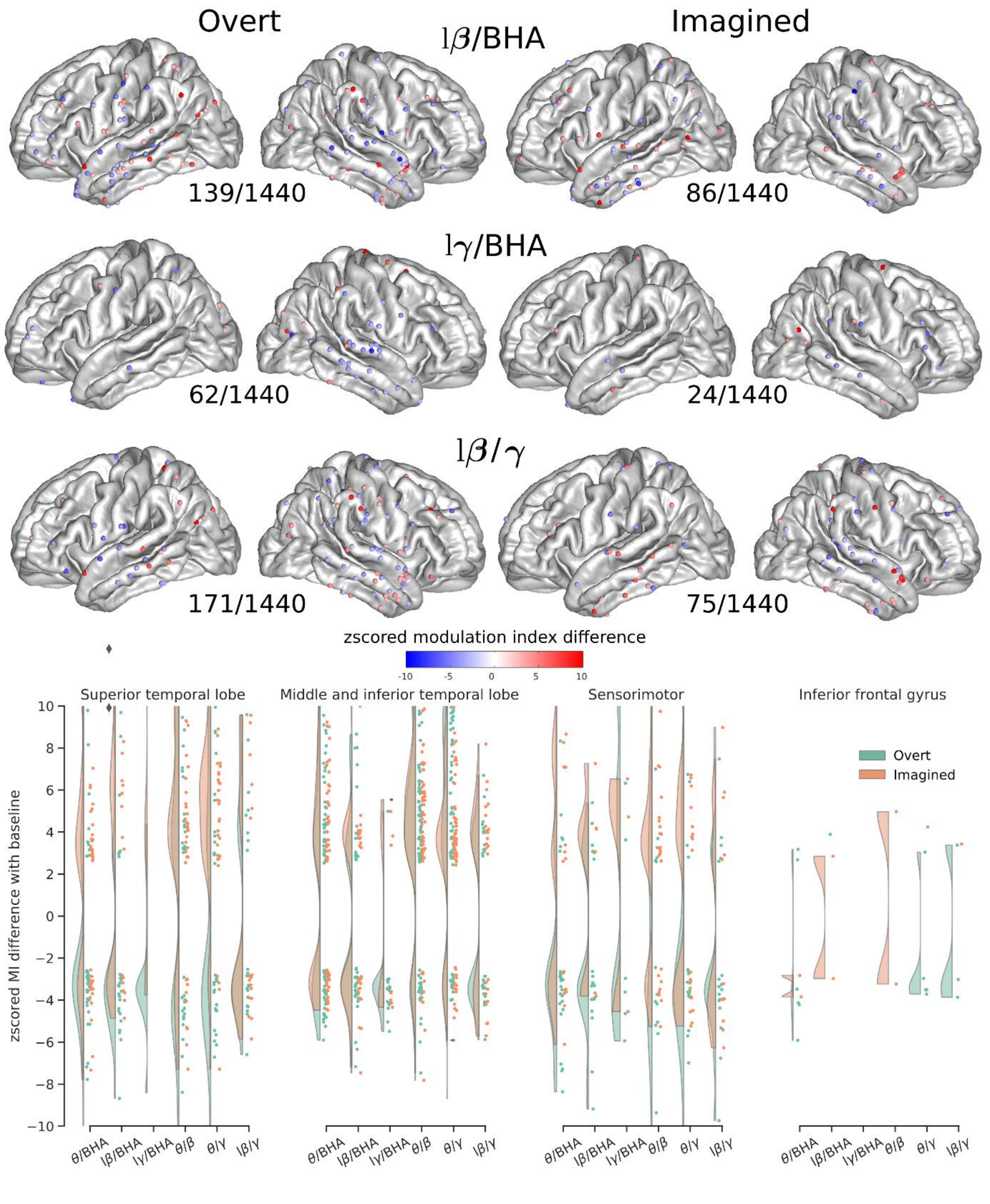

Supplementary Figure 5: Cross-frequency coupling (CFC) between the phase of one frequency band and the amplitude of another frequency band in the same contact. Top: Z-scored modulation index difference for significant electrodes across all participants and studies during overt and imagined speech with respect to baseline (only significant electrodes are shown, permutation tests, FDR-corrected, target threshold α = 0.05). The number of significant electrodes over the total number of electrodes is indicated below each plot. Left column: overt speech. Right column: imagined speech. Results are pulled across all studies, results for separated studies as shown in Supp. Fig 6-8. Bottom: Z-scored modulation index for each ROI and frequency band. Source data are provided as a Source Data file.

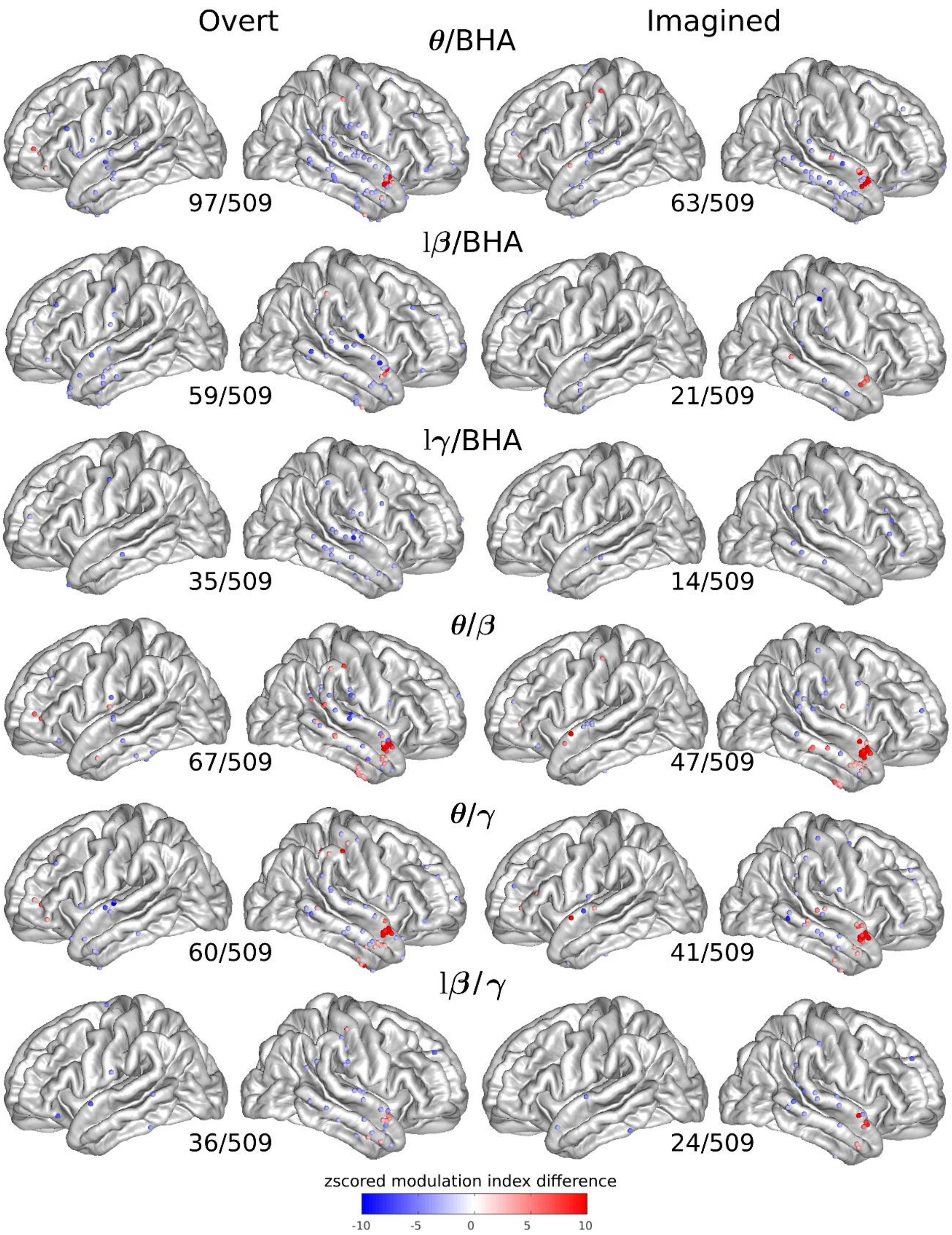

Supplementary Figure 6: Cross-frequency coupling (CFC) between the phase of one frequency band and the amplitude of another frequency band in the same contact for study 1. Z-scored modulation index difference for significant electrodes across participants of study 1 during overt and imagined speech with respect to baseline (only significant electrodes are shown, permutation tests, FDR-corrected, target threshold α = 0.05). The number of significant electrodes over the total number of electrodes is indicated below each plot. Left column: overt speech. Right column: imagined speech.

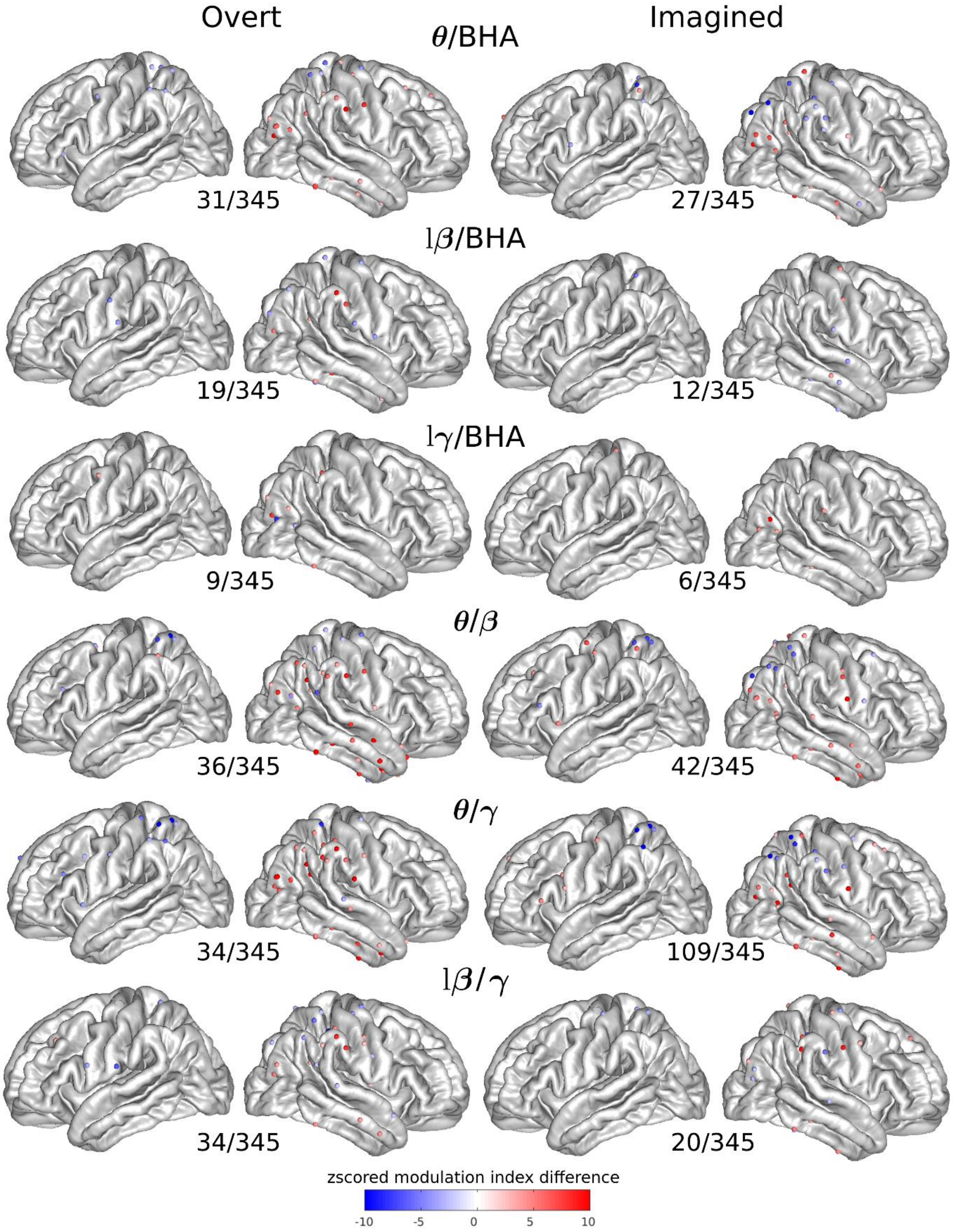

Supplementary Figure 7: Cross-frequency coupling (CFC) between the phase of one frequency band and the amplitude of another frequency band in the same contact for study 2. Z-scored modulation index difference for significant electrodes across participants of study 2 during overt and imagined speech with respect to baseline (only significant electrodes are shown, permutation tests, FDR-corrected, target threshold α = 0.05). The number of significant electrodes over the total number of electrodes is indicated below each plot. Left column: overt speech. Right column: imagined speech.

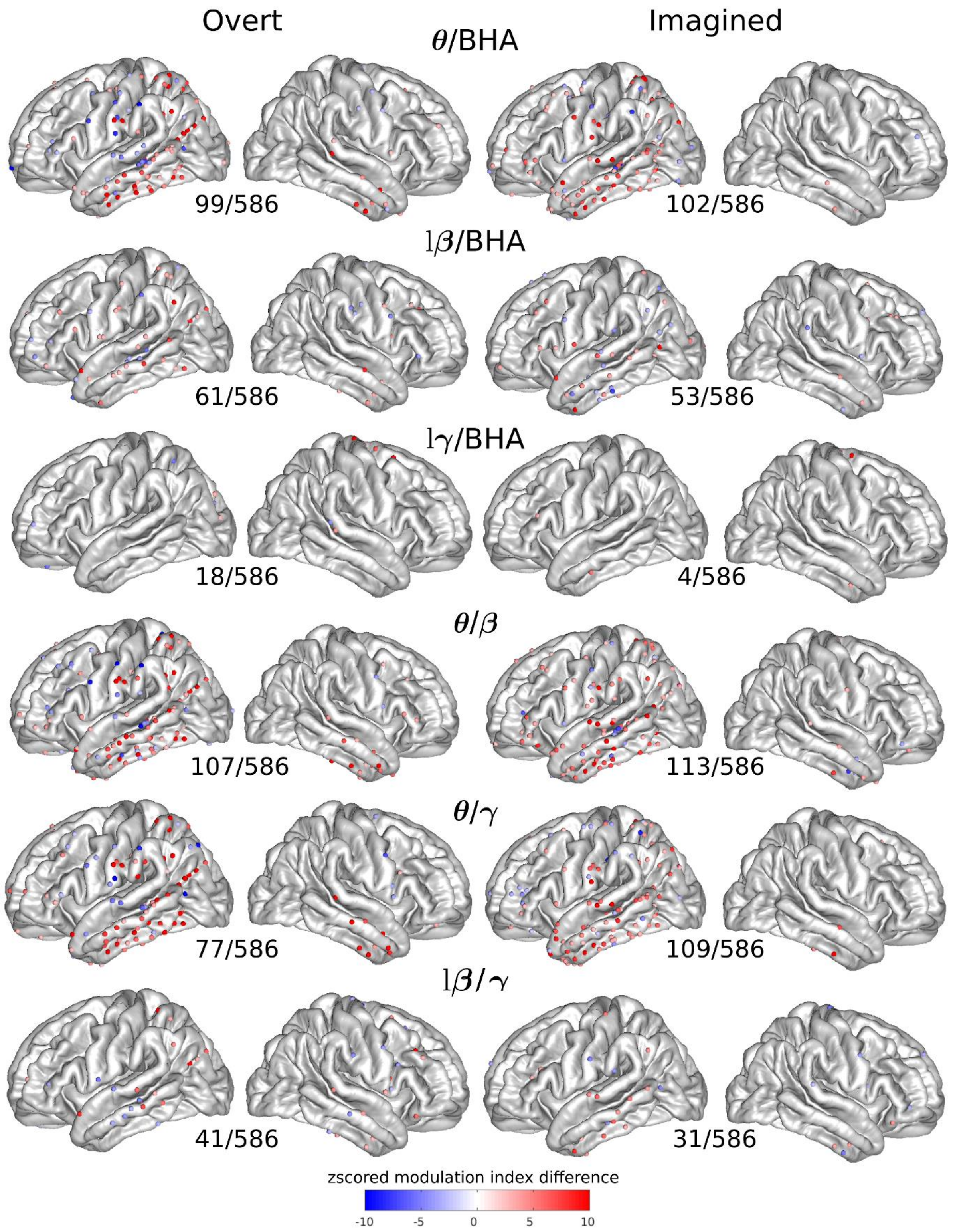

Supplementary Figure 8: Cross-frequency coupling (CFC) between the phase of one frequency band and the amplitude of another frequency band in the same contact for study 3. Z-scored modulation index difference for significant electrodes across participants of study 3 during overt and imagined speech with respect to baseline (only significant electrodes are shown, permutation tests, FDR-corrected, target threshold α = 0.05). The number of significant electrodes over the total number of electrodes is indicated below each plot. Left column: overt speech. Right column: imagined speech.

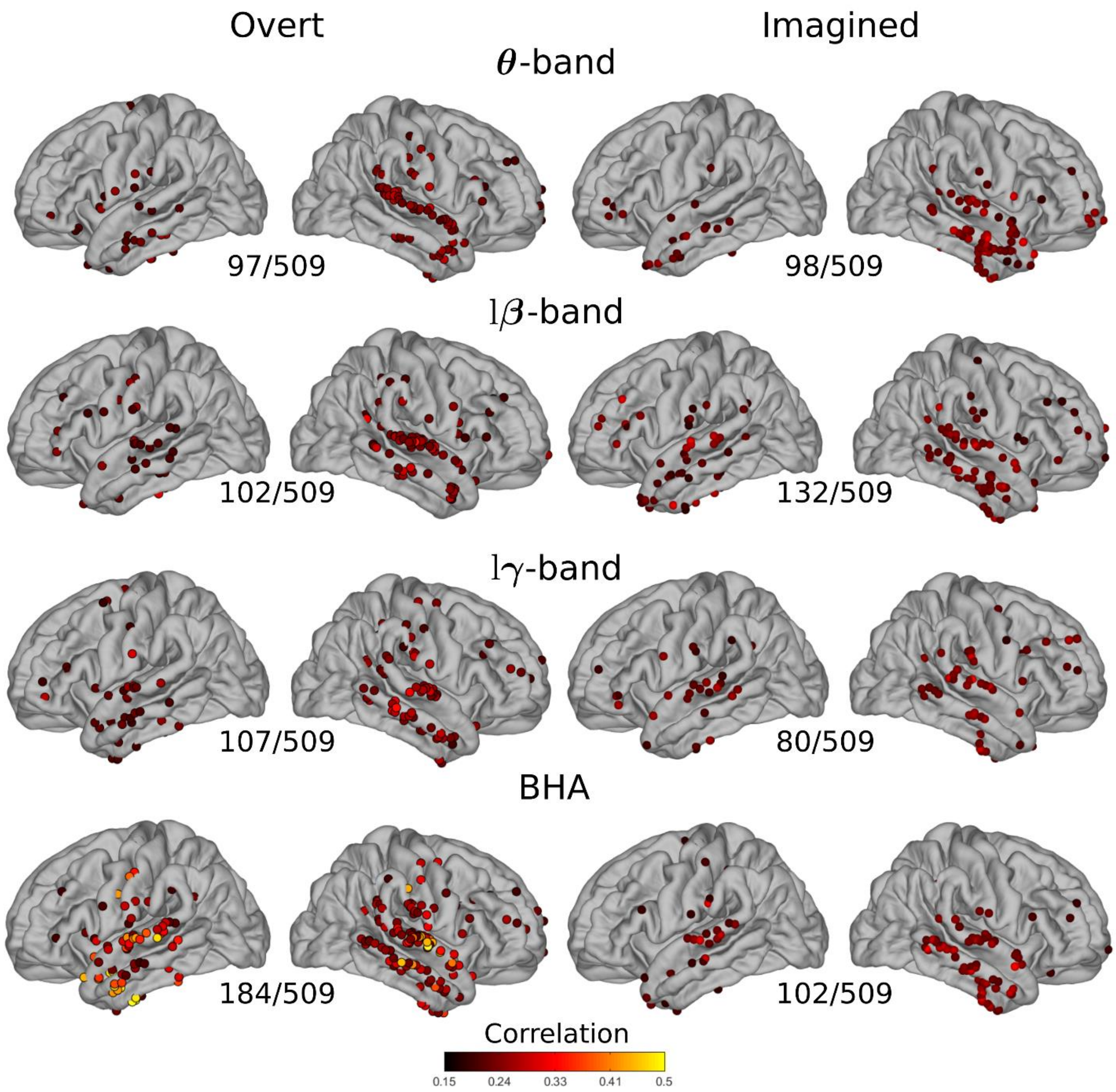

Supplementary Figure 9: Average correlations between individual speech words and their neural representations for study 1. Pairwise correlations between words and power spectrum features averaged across all word pairs of study 1 for overt and imagined speech on significant electrodes (only significant electrodes are shown, permutation tests, $p<0.05$, not corrected for multiple comparison). The number of significant electrodes over the total number of electrodes is indicated below each plot. Left column: overt speech. Right column: imagined speech.

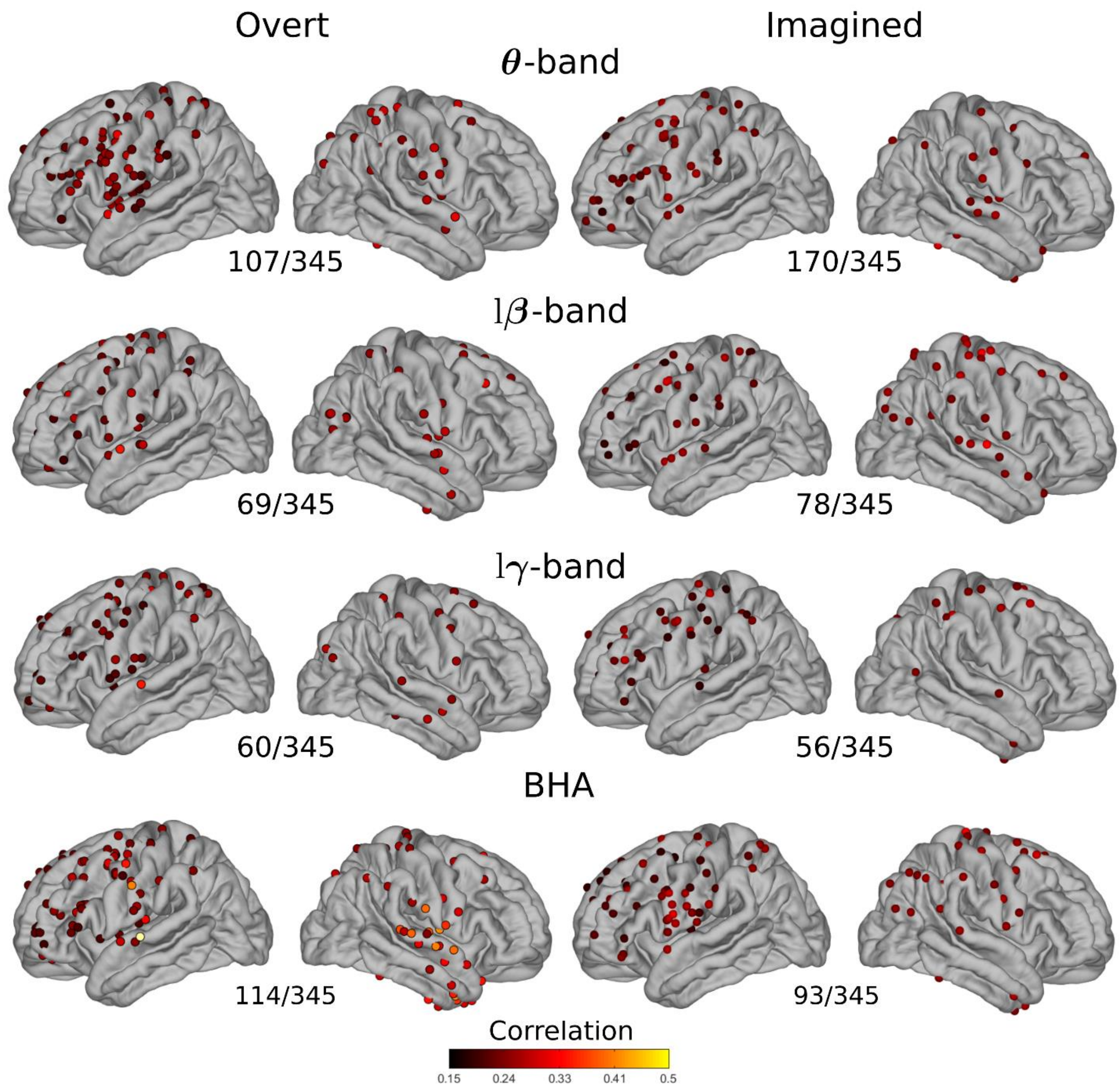

Supplementary Figure 10: Average correlations between individual speech words and their neural representations for study 2. Pairwise correlations between words and power spectrum features averaged across all word pairs of study 2 for overt and imagined speech on significant electrodes (only significant electrodes are shown, permutation tests, $p<0.05$, not corrected for multiple comparison). The number of significant electrodes over the total number of electrodes is indicated below each plot. Left column: overt speech. Right column: imagined speech.

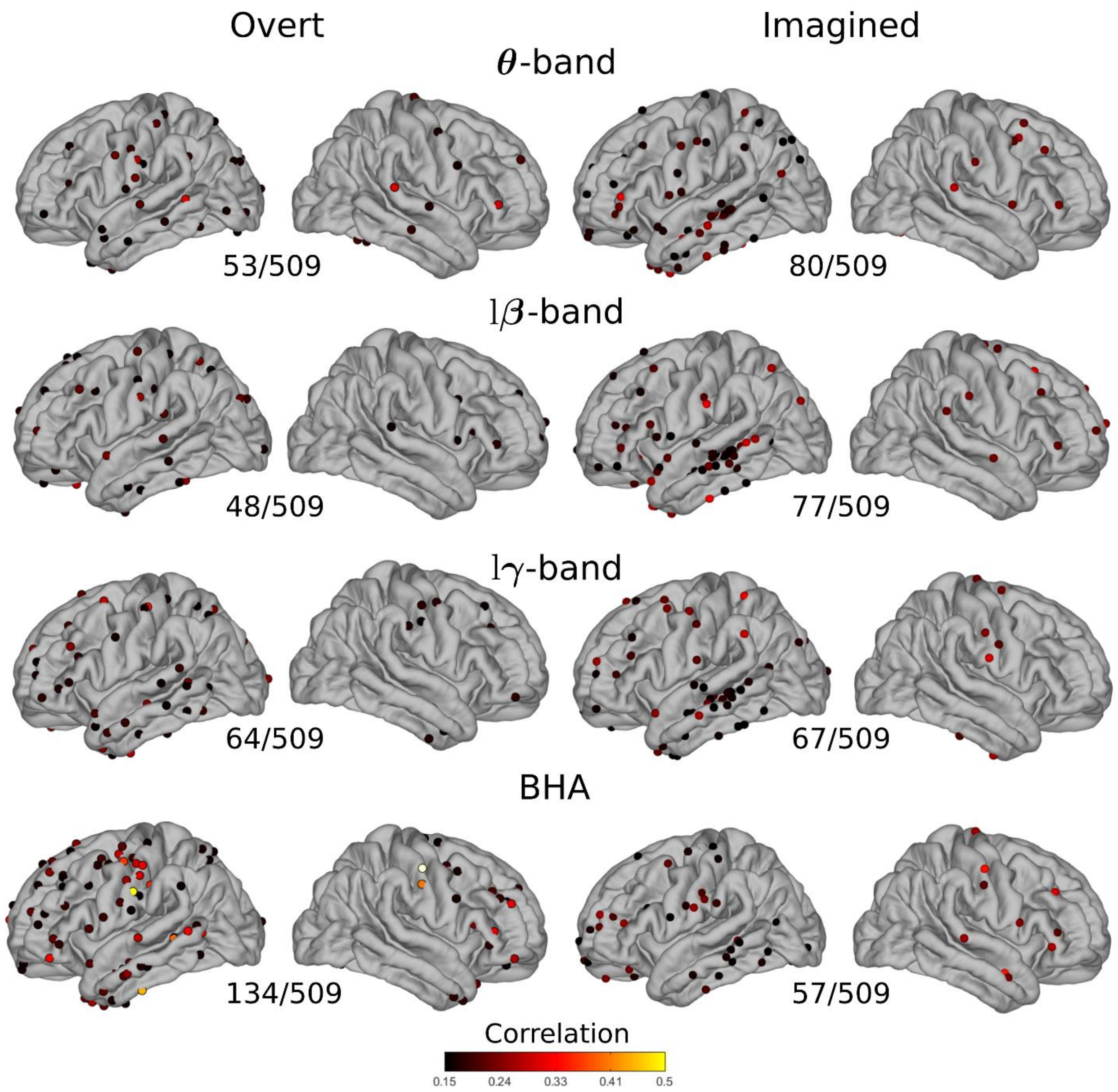

Supplementary Figure 11: Average correlations between individual speech words and their neural representations for study 3. Pairwise correlations between words and power spectrum features averaged across all word pairs of study 3 for overt and imagined speech on significant electrodes (only significant electrodes are shown, permutation tests, $p<0.05$, not corrected for multiple comparison). The number of significant electrodes over the total number of electrodes is indicated below each plot. Left column: overt speech. Right column: imagined speech.

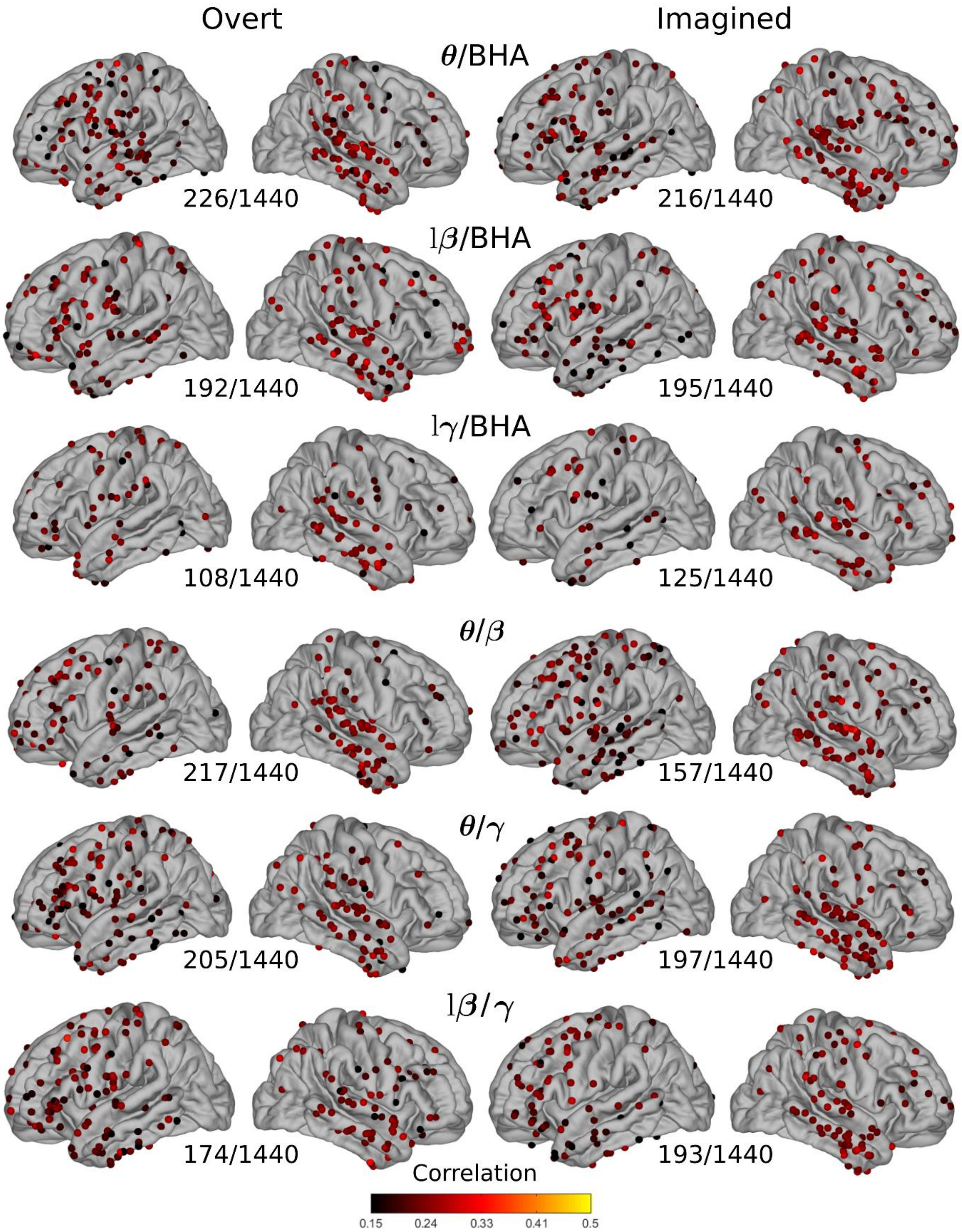

Supplementary Figure 12: Average correlations between individual speech words and phase-amplitude CFC features. Pairwise correlations between words and phase-amplitude CFC features averaged across all pairs for overt and imagined speech on significant electrodes (only significant electrodes are shown, permutation tests, $p<0.05$, not corrected for multiple comparison). The number of significant electrodes over the total number of electrodes is indicated below each plot. Left column: overt speech. Right column: imagined speech. Results are pulled across all studies, results for separated studies as shown in Supp. Fig 13-15.

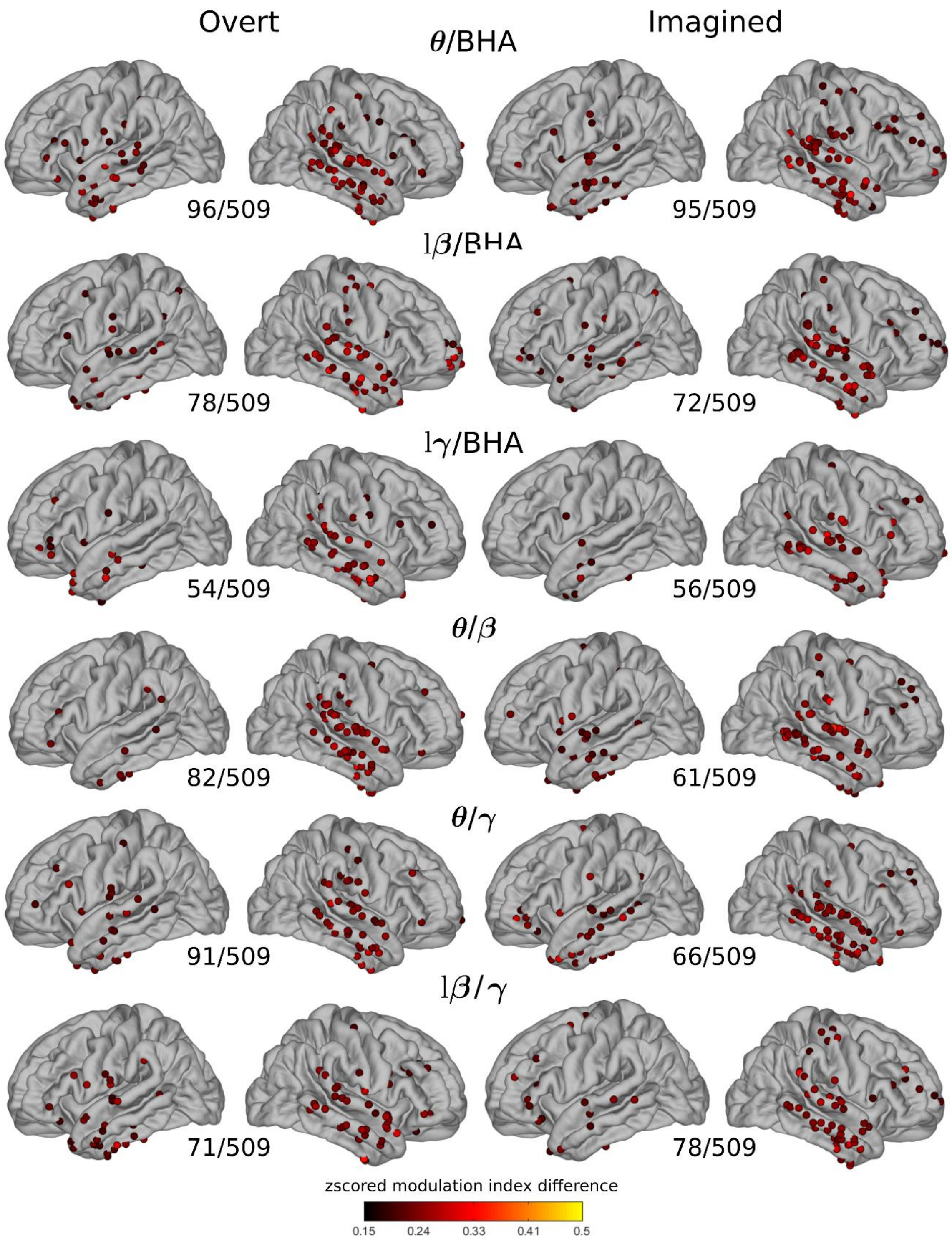

Supplementary Figure 13: Average correlations between individual speech words and phase-amplitude CFC features for study 1. Pairwise correlations between words and phase-amplitude CFC features averaged across all pairs of study 1 for overt and imagined speech on significant electrodes (only significant electrodes are shown, permutation tests, $p<0.05$, not corrected for multiple comparison). The number of significant electrodes over the total number of electrodes is indicated below each plot. Left column: overt speech. Right column: imagined speech.

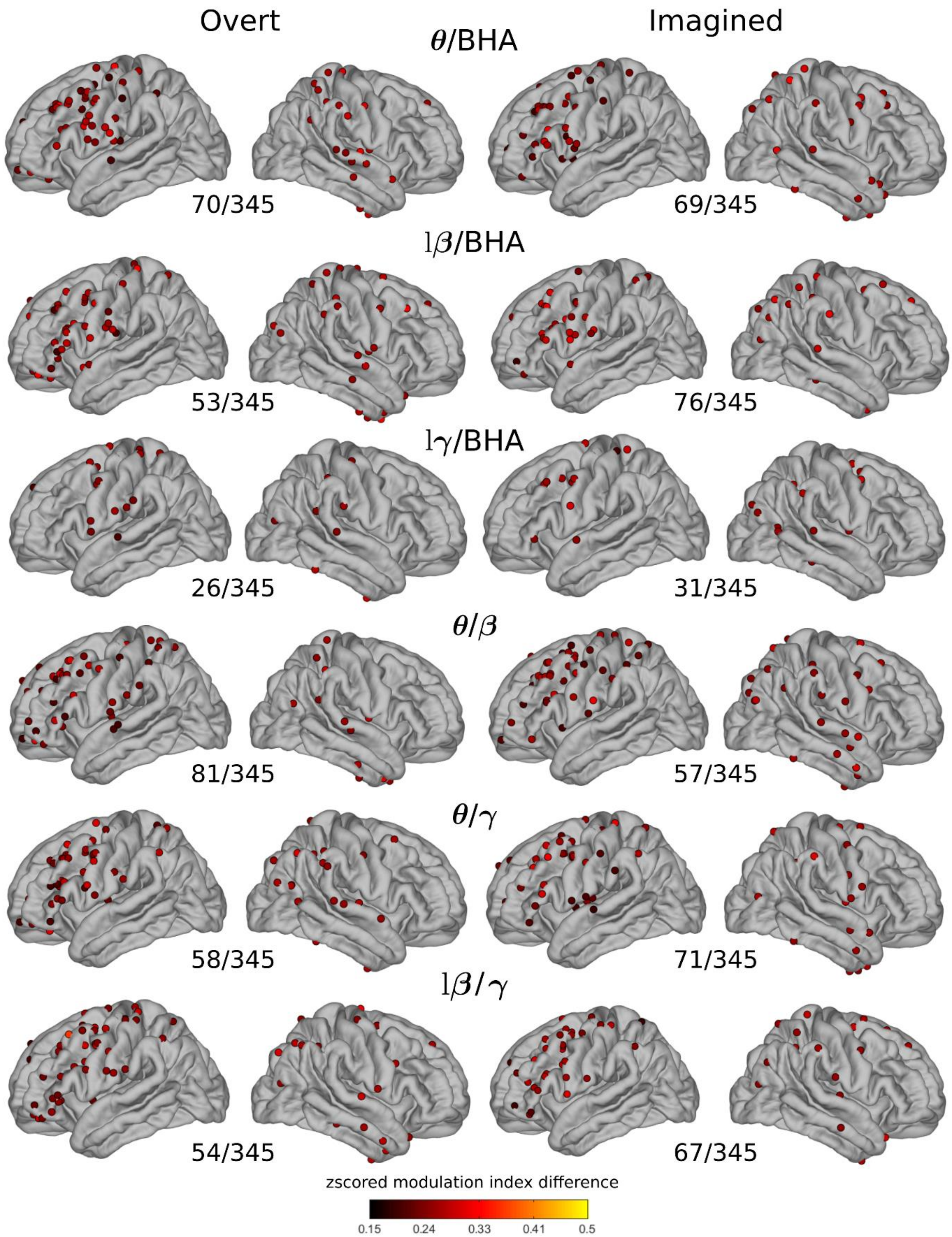

Supplementary Figure 14: Average correlations between individual speech words and phase-amplitude CFC features for study 2. Pairwise correlations between words and phase-amplitude CFC features averaged across all pairs of study 2 for overt and imagined speech on significant electrodes (only significant electrodes are shown, permutation tests, $p<0.05$, not corrected for multiple comparison). The number of significant electrodes over the total number of electrodes is indicated below each plot. Left column: overt speech. Right column: imagined speech.

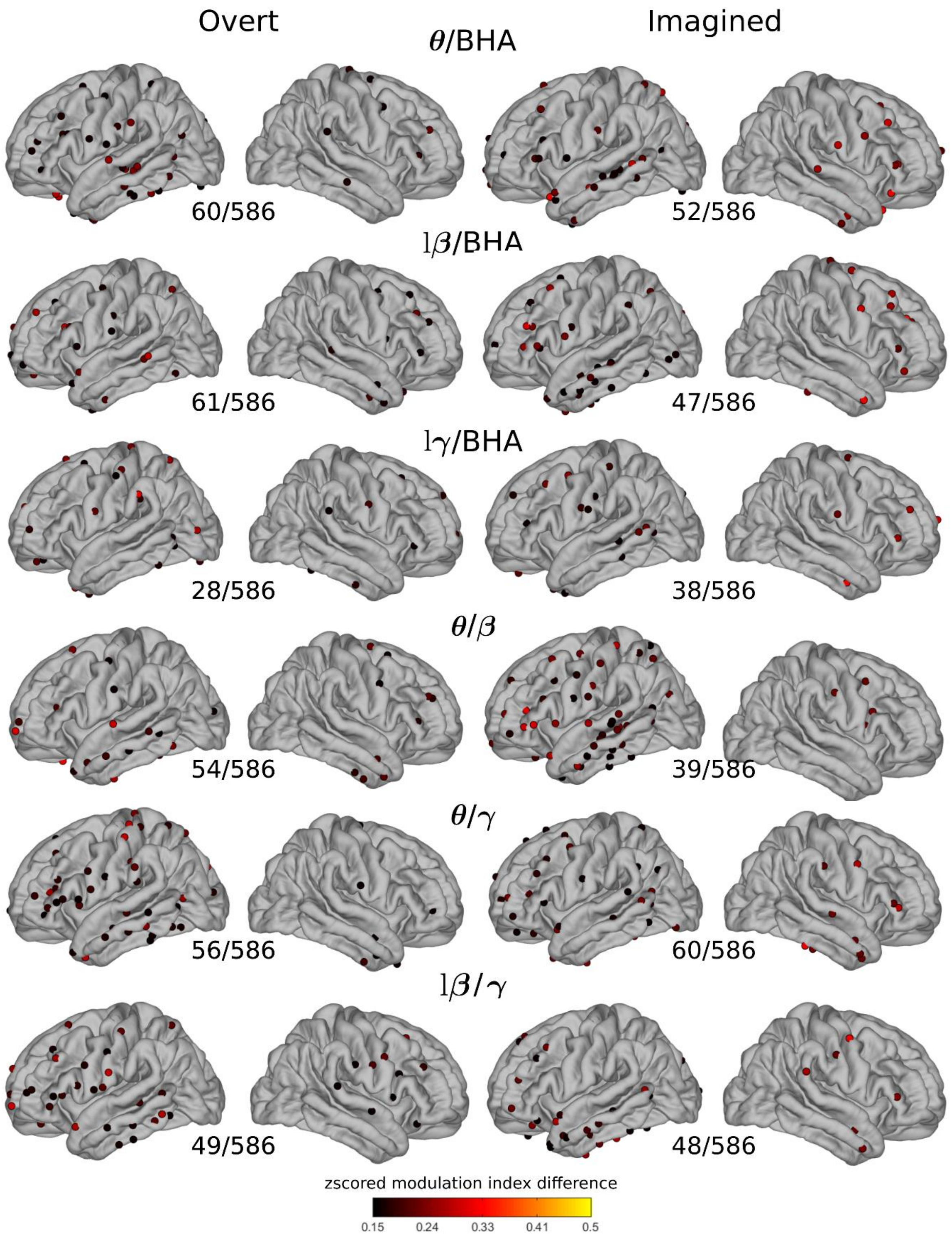

Supplementary Figure 15: Average correlations between individual speech words and phase-amplitude CFC features for study 3. Pairwise correlations between words and phase-amplitude CFC features averaged across all pairs of study 3 for overt and imagined speech on significant electrodes (only significant electrodes are shown, permutation tests, $p<0.05$, not corrected for multiple comparison). The number of significant electrodes over the total number of electrodes is indicated below each plot. Left column: overt speech. Right column: imagined speech.

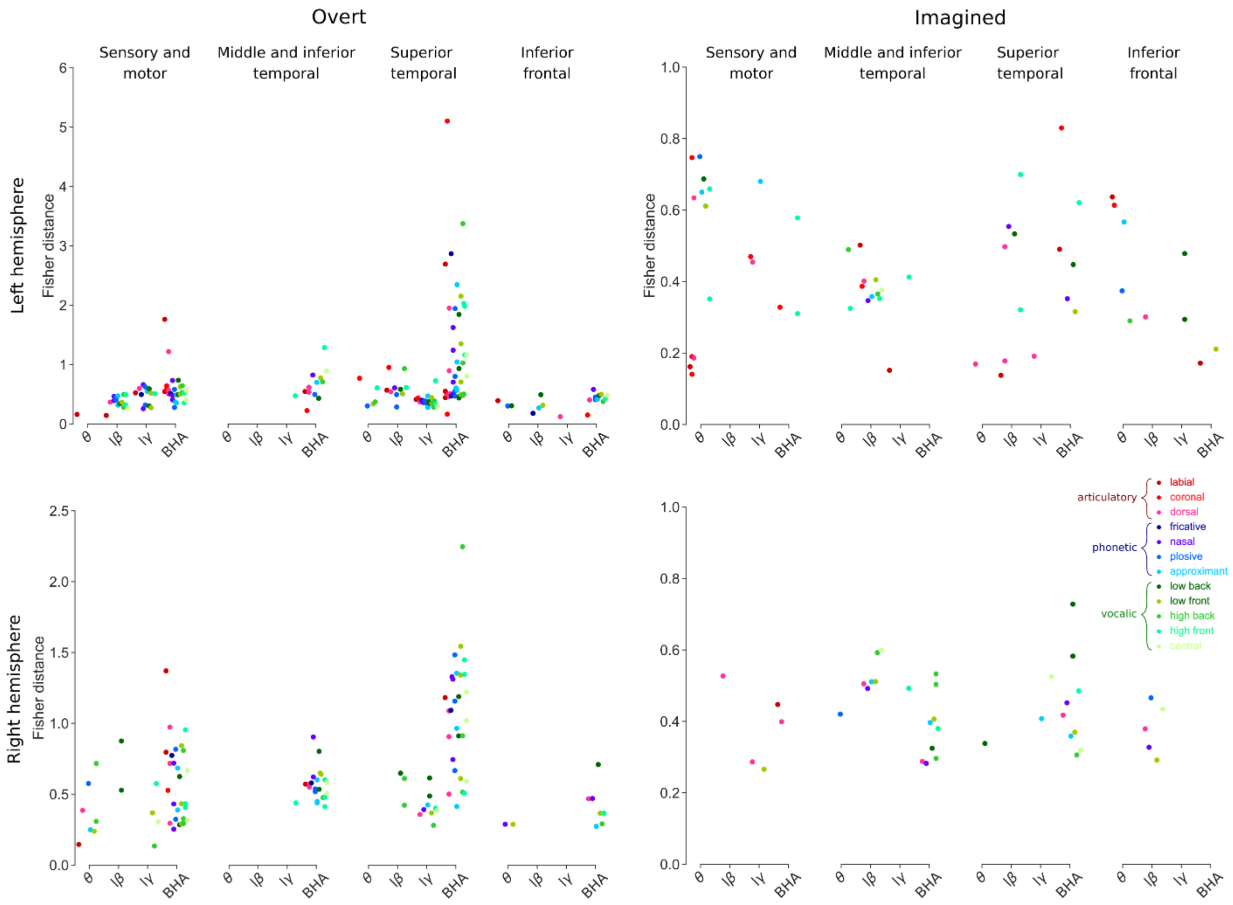

Supplementary Figure 16: Separation of power spectrum changes for articulatory, phonetic and vowel representations in different brain regions and frequency bands. Only significant values are shown (permutation test, FDR corrected, target threshold α = 0.05). Note the different scales between overt and imagined speech. Source data are provided as a Source Data file.

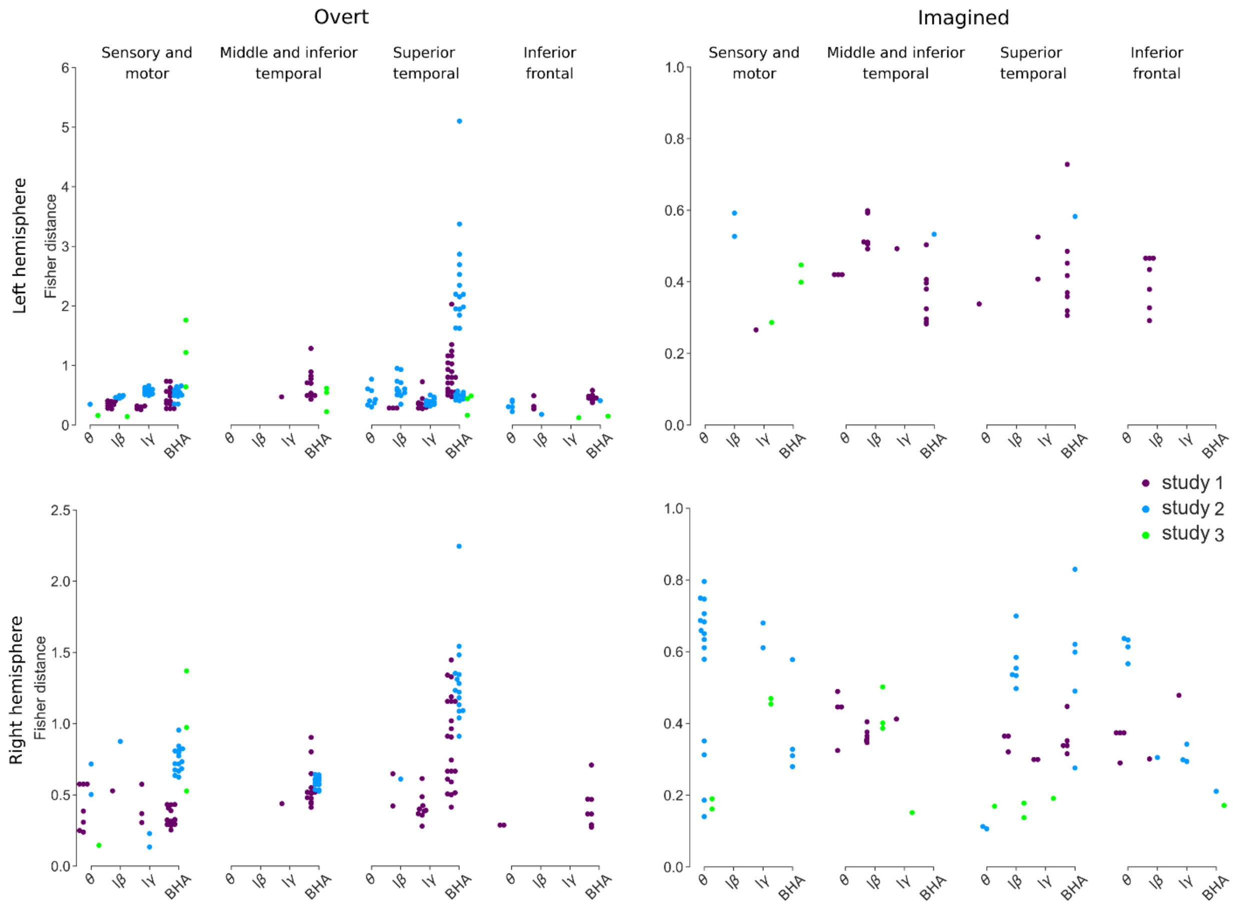

Supplementary Figure 17: Separation of power spectrum changes for each study in different brain regions and frequency bands. Only significant values are shown (permutation test, FDR corrected, target threshold $\alpha = 0.05$). Note the different scales between overt and imagined speech. Source data are provided as a Source Data file.

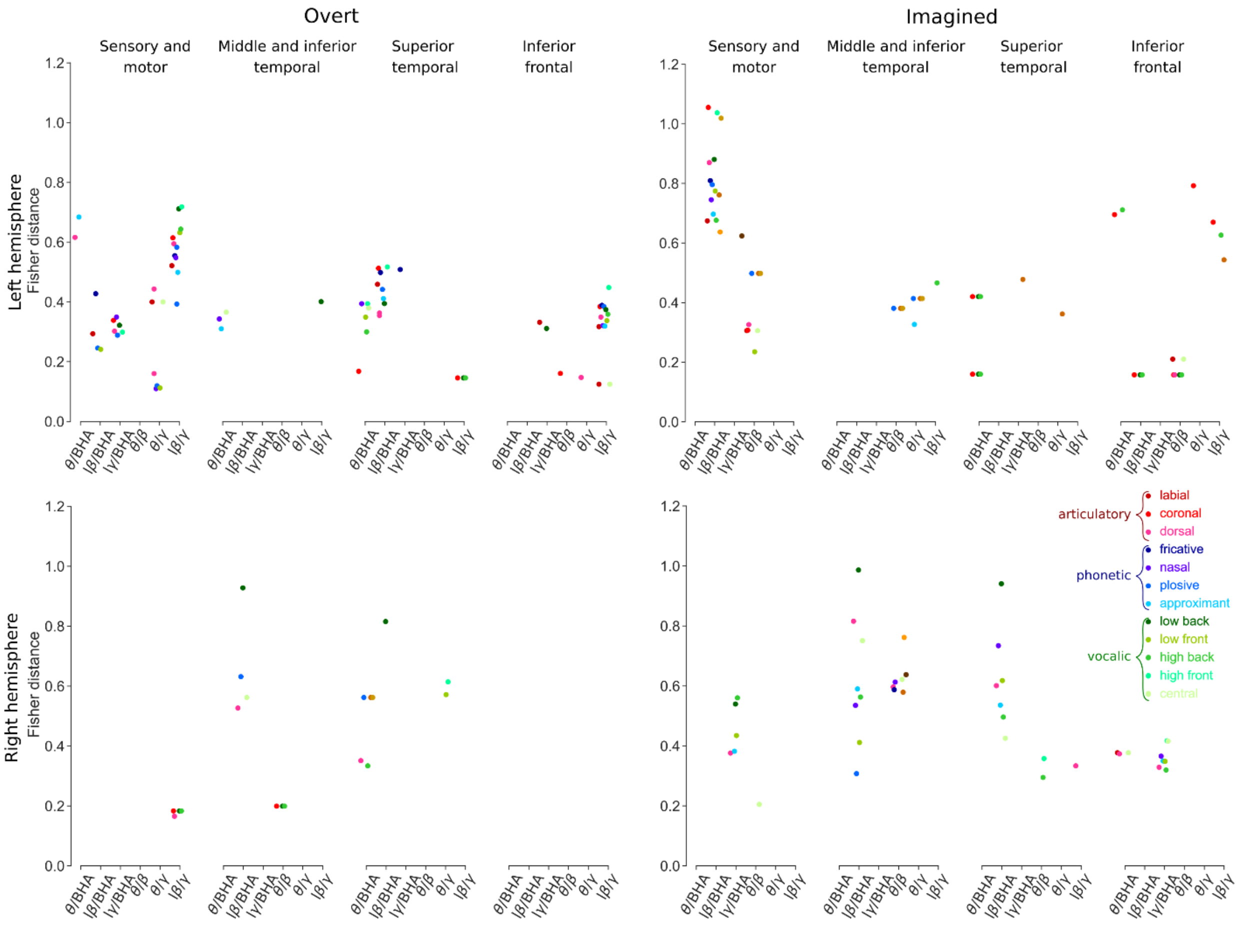

Supplementary Figure 18: Separation of phase-amplitude CFC changes for articulatory, phonetic and vowel representations in different brain regions and frequency bands. Only significant values are shown (permutation test, FDR corrected, target threshold $\alpha = 0.05$). Source data are provided as a Source Data file.

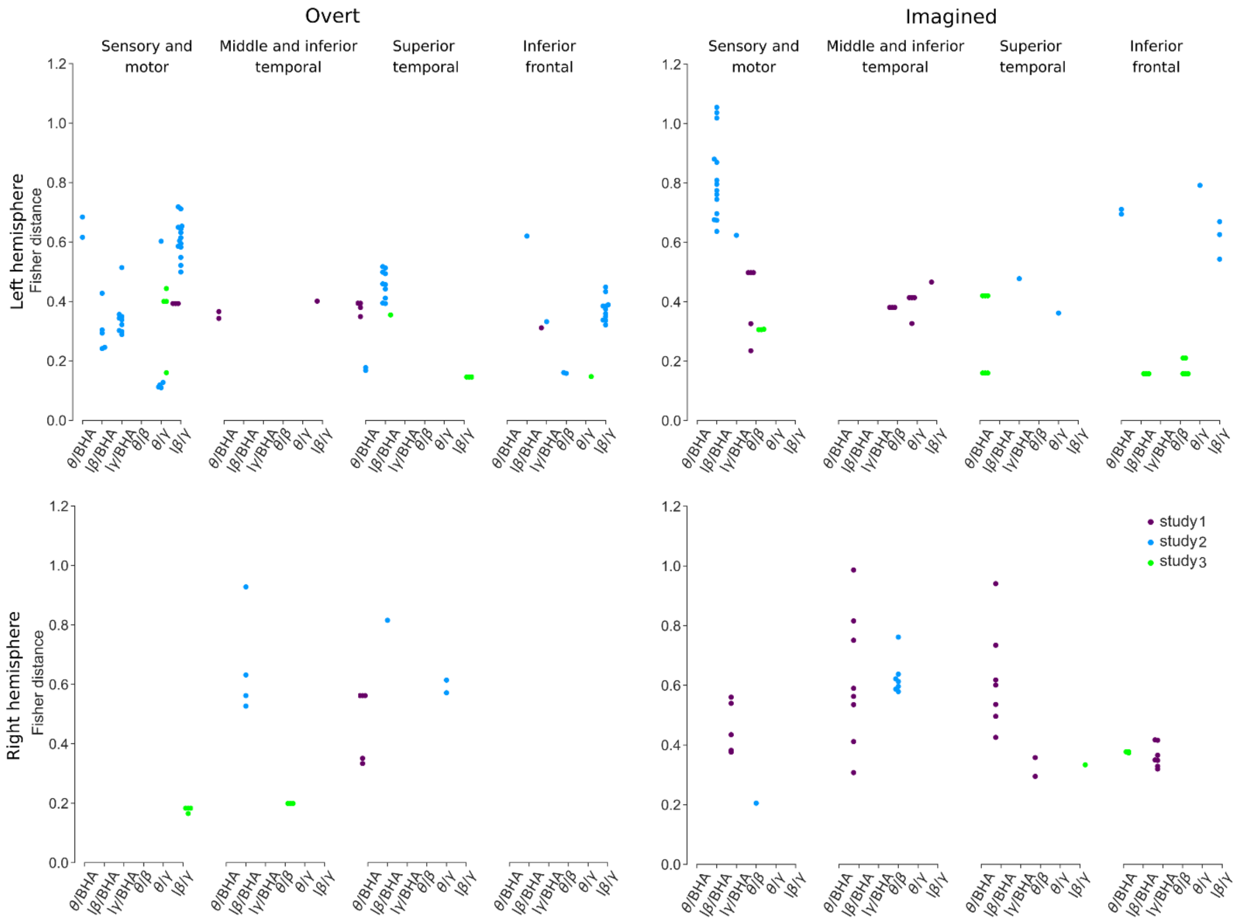

Supplementary Figure 19: Separation of phase-amplitude CFC changes for each study in different brain regions and frequency bands. Only significant values are shown (permutation test, FDR corrected, target threshold α = 0.05). Source data are provided as a Source Data file.

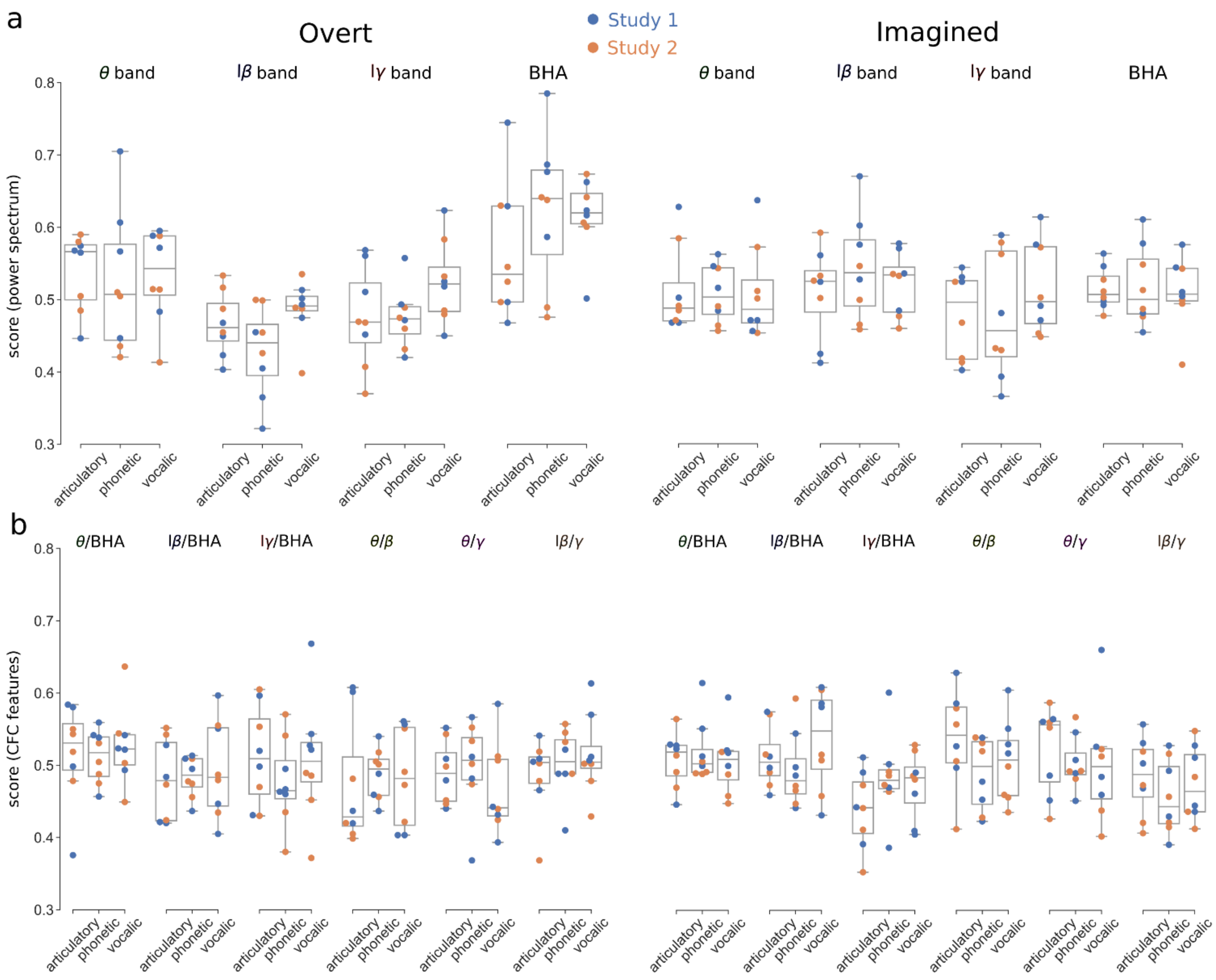

Supplementary Figure 20: Decoding overt (left) and imagined (right) speech for study 1 and 2 (N=8, studies 1 and 2). Boxplots' center, bound of box, and whiskers show respectively the median, interquartile range, and the extent of the distribution (outliers excepted). (**a**) Decoding performance using power spectrum features. (**b**) Decoding performance using phase-amplitude CFC features. Source data are provided as a Source Data file.

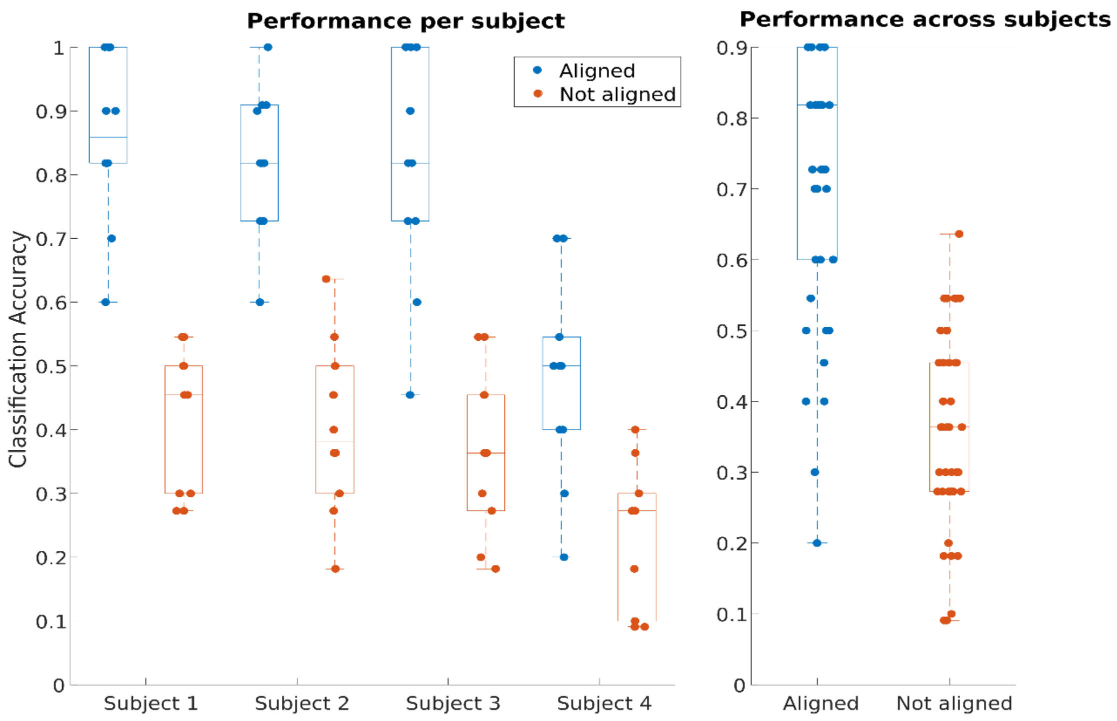

Supplementary Figure 21: Decoding performance of overt speech increases significantly after realigning trials for study 1. Multiclass classification results (6 classes). Trials were aligned on the onset of audio recordings during production. A linear discriminant function was trained using the electrodes that show activity during the task with respect to baseline. Classification performance was obtained using a stratified 10-fold cross-validation, using each time 90% of the data for training and 10% for testing. Only subjects of study 1 were used (N=4). Left, for each subject separately (N=10 folds). Right, across all subjects (N=40). Boxplots' center, bound of box, and whiskers show respectively the median, interquartile range, and the extent of the distribution. Source data are provided as a Source Data file.

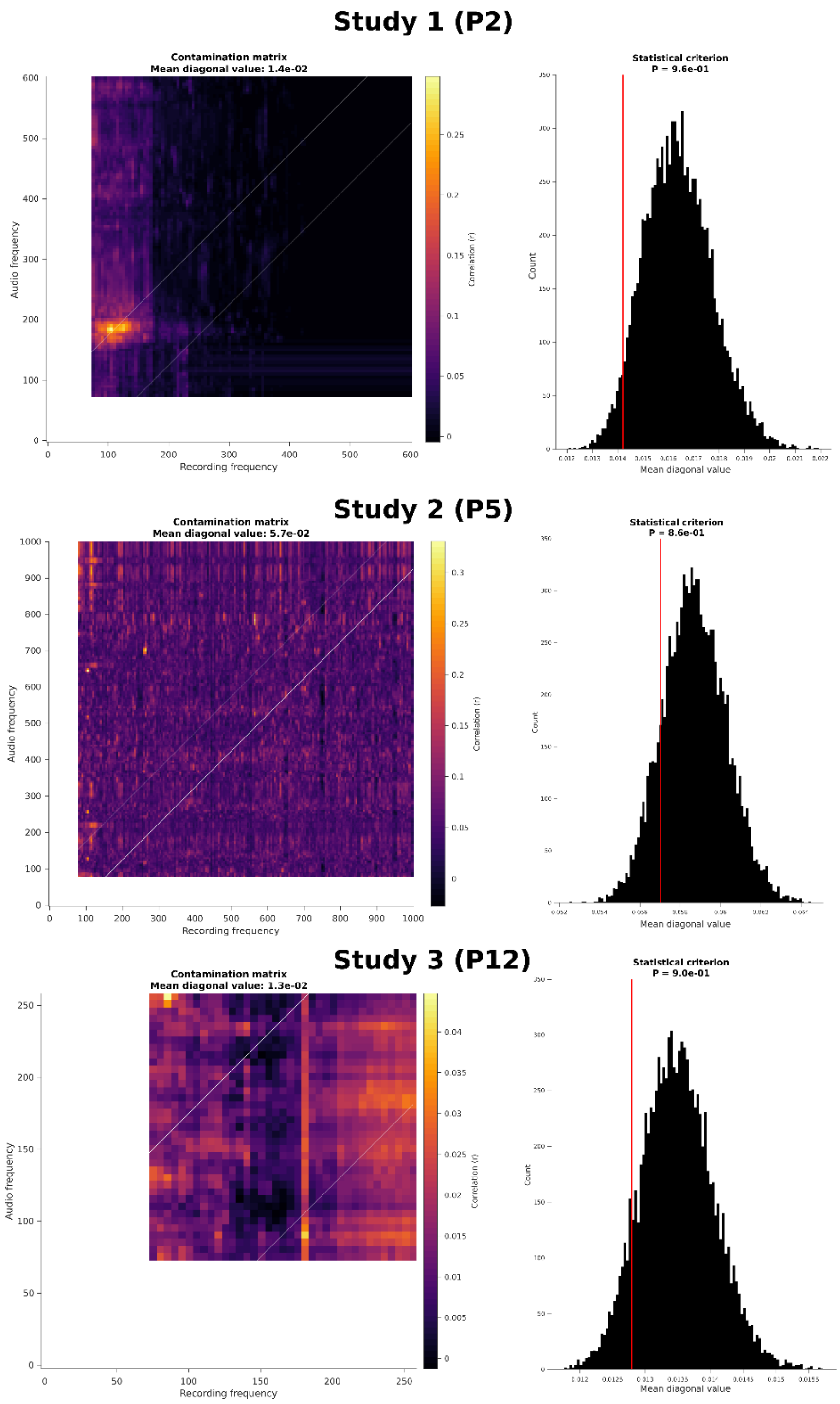

Supplementary Figure 22: Contamination matrices and statistical assessment of contamination for one patient of each dataset. Left column: audio-neural contamination matrices for the three studies. Each heatmap corresponds to a patient's recording. Frequencies below 75 Hz were discarded to avoid influence from 50 Hz and 60 Hz power noise. White lines help assessing presence of high correlations in the diagonal of each matrix. Right column: statistical assessment of contamination for the three corresponding studies. Mean value of the diagonal is compared to a null distribution obtained by booster (10.000 shuffled versions of the contamination matrix). Code used to generate this figure is provided with Roussel et al., 2020, J. Neural Eng. 17(5). The analysis was repeated for all patients, without finding significant effects.

Power	Overt								Covert							
Representation	Articulatory				Perceptual				Articulatory				perceptual			
Band	θ	lβ	lγ	BHA	θ	lβ	lγ	BHA	θ	lβ	lγ	BHA	θ	lβ	lγ	BHA
Sensory and motor (p-values*10^2)	1.5<br>1.6<br>2.3<br>0.3	0.4<br>1.1	0.3<br>2.6<br>3.2<br>2.1<br>1.4	0.0<br>0.7<br>0.0<br>0.4<br>2.4<br>0.5<br>0.0	NA	2.0<br>1.5	0.1	4.9<br>0.0<br>0.0<br>0.0<br>0.2<br>0.0<br>0.0	2.1<br>0.1	3.1<br>4.9<br>0.9<br>0.7	3.8<br>1.8	0.0<br>0.4<br>0.1	3.3	0.7	4.2	3.9
Middle and inferior temporal (p-values*10^2)	2.0	NA	2.4<br>4.8	0.0<br>2.8<br>0.1	NA	0.1	NA	0.0<br>0.0<br>0.1	3.6	0.5	NA	NA	NA	1.2	3.2	NA
Superior temporal (p-values*10^2)	0.2	4.0	4.7<br>2.3<br>0.0	0.0<br>0.0<br>3.1<br>3.8<br>3.8	0.4<br>2.0	0.3<br>0.5<br>3.6<br>4.0	0.5<br>2.2	0.0<br>0.0<br>0.0<br>0.0<br>0.0<br>0.0	0.7<br>4.0	1.8<br>1.4<br>0.3<br>1.4	2.4<br>0.2<br>3.6	0.4	NA	2.2<br>4.1<br>2.8	0.3<br>1.6	3.5
Inferior frontal (p-values*10^2)	4.3	4.9	3.0	2.4<br>1.9	4.5	NA	NA	1.2	2.8	NA	NA	NA	1.7	NA	NA	NA
Total of significant Fisher distances	7	4	11	17	3	7	3	17	6	9	5	4	2	5	4	2

Supplementary Table 1: P-values and number of significant Fisher distances for power spectrum across subjects. Multi-way ANOVA, $p<0.05$, no FDR correction.

Phase-amplitude CFC	Overt											
Representation	Articulatory						Perceptual					
Band	θ/BHA	lβ/BHA	lγ/BHA	θ/ lβ	θ/lγ	lβ/ lγ	θ/BHA	lβ/BHA	lγ/BHA	θ/ lβ	θ/lγ	lβ/ lγ
Sensory and motor (p-values*10^2)	2.0	4.1	NA	NA	NA	NA	0.1	NA	NA	NA	NA	NA
Middle and inferior temporal (p-values*10^2)	4.7<br>4.1	NA	0.1	2.0<br>3.9<br>3.8<br>0.6	3.8	1.2<br>3.6	NA	0.5	3.9	NA	NA	1.7<br>3.5
Superior temporal (p-values*10^2)	4.8	1.8	4.7	NA	1.9<br>3.4	NA	0.8	1.6	NA	NA	NA	NA
Inferior frontal (p-values*10^2)	NA	NA	NA	NA	NA	NA	NA	NA	NA	NA	NA	NA
Total	4	2	2	4	3	2	2	2	1	0	0	2
	Covert											
Sensory and motor (p-values*10^2)	NA	NA	NA	2.3	NA	1.5<br>3.1	NA	NA	NA	2.0	1.2	NA
Middle and inferior temporal (p-values*10^2)	NA	4.1<br>1.7<br>4.5<br>0.4	NA	NA	3.5	1.4<br>1.7	2.2<br>0.7	NA	2.5	0.1	NA	4.0
Superior temporal (p-values*10^2)	1.4	NA	1.5	1.9<br>0.2	3.0	NA	3.0	NA	NA	NA	NA	NA
Inferior frontal (p-values*10^2)	NA	NA	NA	1.2	NA	NA	NA	NA	NA	NA	NA	NA
Total	1	4	1	4	2	4	3	0	1	2	1	1

Supplementary Table 2: P-values and number of significant Fisher distances for phase-amplitude CFC across subjects. Multi-way ANOVA, $p<0.05$, no FDR correction.

Study	Age	Sex	Handedness	Speech lateralization	Number of recording contacts
Study1	19-33 (1 NA)	4 female	2L, 2R	2L, 2R (iEEG)	79-294
Study 2	20-49	3 female, 1 male	2L, 2R	3L, 1R (fMRI1 ESM)	32-124
Study 3	23-42	3 female, 2 male	5R	3L, 1 bilateral, 1unknown	120-125

Supplementary Table 3: Clinical information for participants in the three studies. L: left, R: right.

	Phoneme																					
	b	d	f	g	k	l	m	n	p	K	s	t	v	z	T	N	j	z	S	¸c	r	w
**Articulatory**																						
Labial	x		x				x		x				x									
Coronal		x				x		x			x	x		x	x			x	x		x	
Dorsal				x	x					x						x	x			x		x
**Phonetic**																						
Nasal							x	x								x						
Plosive	x	x		x	x				x			x										
Approximant						x											x					x

Supplementary Table 4: Articulatory and phonetic representation for each phoneme used in the tasks.

	Phoneme														
	i	y	I	Y	e	E	æ	œ	a	@	u	U	O	˜O	A˜
**Vocalic**															
Front	x	x	x	x	x	x	x	x	x						
Middle										x					
Back											x	x	x	x	x
High	x	x	x	x	x						x	x			
Central										x					
Low						x	x	x	x				x	x	x

Supplementary Table 5: Vocalic representation for each vowel used in the tasks.
