# Supplementary material for: Imagined speech can be decoded from low- and cross-frequency intracranial EEG features
Source: Nat Commun. 2022 Jan 10;13:48. doi: 10.1038/s41467-021-27725-3 (PMC8748882; doi:10.1038/s41467-021-27725-3)
Supplement: Supplementary file 3 — Reporting Summary [file 41467_2021_27725_MOESM3_ESM.pdf]

nature portfolio

Corresponding author(s): Timothée Proix

Last updated by author(s): Nov 5, 2021

Reporting Summary

Nature Portfolio wishes to improve the reproducibility of the work that we publish. This form provides structure for consistency and transparency in reporting. For further information on Nature Portfolio policies, see our Editorial Policies and the Editorial Policy Checklist.

Statistics

For all statistical analyses, confirm that the following items are present in the figure legend, table legend, main text, or Methods section.

n/a	Confirmed	
☐	☒	The exact sample size (*n*) for each experimental group/condition, given as a discrete number and unit of measurement
☐	☒	A statement on whether measurements were taken from distinct samples or whether the same sample was measured repeatedly
☐	☒	The statistical test(s) used AND whether they are one- or two-sided<br>*Only common tests should be described solely by name; describe more complex techniques in the Methods section.*
☐	☒	A description of all covariates tested
☐	☒	A description of any assumptions or corrections, such as tests of normality and adjustment for multiple comparisons
☐	☒	A full description of the statistical parameters including central tendency (e.g. means) or other basic estimates (e.g. regression coefficient) AND variation (e.g. standard deviation) or associated estimates of uncertainty (e.g. confidence intervals)
☐	☒	For null hypothesis testing, the test statistic (e.g. *F*, *t*, *r*) with confidence intervals, effect sizes, degrees of freedom and *P* value noted<br>*Give P values as exact values whenever suitable.*
☒	☐	For Bayesian analysis, information on the choice of priors and Markov chain Monte Carlo settings
☒	☐	For hierarchical and complex designs, identification of the appropriate level for tests and full reporting of outcomes
☐	☒	Estimates of effect sizes (e.g. Cohen's *d*, Pearson's *r*), indicating how they were calculated

Our web collection on statistics for biologists contains articles on many of the points above.

Software and code

Policy information about availability of computer code

Data collection	Data collection was made using clinical software used in the three hospitals were the experiments were run. Stimuli presentation was designed with Psychotoolbox-3, BCI2000 version 3 and Presentation 14.
Data analysis	Software:<br>- Matlab R2018b<br>- Python 3.8.5<br>Custom code was created for the analysis presented in the manuscript and is provided.

For manuscripts utilizing custom algorithms or software that are central to the research but not yet described in published literature, software must be made available to editors and reviewers. We strongly encourage code deposition in a community repository (e.g. GitHub). See the Nature Portfolio guidelines for submitting code & software for further information.

Data

Policy information about availability of data

All manuscripts must include a data availability statement. This statement should provide the following information, where applicable:

- Accession codes, unique identifiers, or web links for publicly available datasets
- A description of any restrictions on data availability
- For clinical datasets or third party data, please ensure that the statement adheres to our policy

Raw patient-related data are protected and are not available due to data privacy laws. Processed neurophysiological and neuroanatomical data are available under restricted access for ethical and privacy reasons. Access to data collected at Geneva University Hospitals can be requested by contacting Pierre Mégevand

(pierre.megevand@unige.ch) and is conditional to the establishment of a specific data sharing agreement between the applicant's institution and the University of Geneva. Source data are provided with this paper. Requests will be handled within a timeframe of three months.

Field-specific reporting

Please select the one below that is the best fit for your research. If you are not sure, read the appropriate sections before making your selection.

☒ Life sciences ☐ Behavioural & social sciences ☐ Ecological, evolutionary & environmental sciences

For a reference copy of the document with all sections, see nature.com/documents/nr-reporting-summary-flat.pdf

Life sciences study design

All studies must disclose on these points even when the disclosure is negative.

Sample size	No sample-size calculation was performed. Intracranial EEG data are rare data, and all relevant data available was used. A total of 1440 ECoG contacts were used, this is more than most published intracranial EEG articles.
Data exclusions	One patient from the first study was excluded, as the patient was whispering words instead of pronouncing them covertly (pre-established exclusion criteria that no sounds are uttered during imagined speech task). This was revealed after listening to the recorded audio of each experiment.
Replication	Each study was repeated in 4 or 5 patients. Results were found to be replicable across patients in each study, in the limit of the different electrode coverages.
Randomization	Allocation of patients to each study was not randomized, but depended on which study center the patient was recruited, as each study center was performing a distinct experiment.
Blinding	No blinding was possible: auditory or visual stimuli were presented to the subject, that he/she had to repeat, and therefore had to be aware of what the stimuli were.

Reporting for specific materials, systems and methods

We require information from authors about some types of materials, experimental systems and methods used in many studies. Here, indicate whether each material, system or method listed is relevant to your study. If you are not sure if a list item applies to your research, read the appropriate section before selecting a response.

Materials & experimental systems

n/a	Involved in the study	
☒	☐	Antibodies
☒	☐	Eukaryotic cell lines
☒	☐	Palaeontology and archaeology
☒	☐	Animals and other organisms
☐	☒	Human research participants
☒	☐	Clinical data
☒	☐	Dual use research of concern

Methods

n/a	Involved in the study	
☒	☐	ChIP-seq
☒	☐	Flow cytometry
☒	☐	MRI-based neuroimaging

Human research participants

Policy information about studies involving human research participants

Population characteristics	Covariate-relevant population characteristics are: age, sex, handedness, and speech lateralization, and are all provided as Supplementary Table 3
Recruitment	Patients with refractory epilepsy using subdural electrode arrays implanted as part of the standard presurgical evaluation process were recruited. All participants gave informed consent. There was no group into which the patient could select themselves, therefore self-selection bias is irrelevant. No other potential biases were identified.
Ethics oversight	Electrocorticographic (ECoG) recordings were obtained in 3 distinct studies from 13 patients (study 1: 4 participants, 4 women, mean age 25.6 years, range 19-33; study 2: 4 participants, 3 women, mean age 30.5 years, range 20-49; study 3: 5 participants, 3 women, mean age 32.6 years, range 23-42) with refractory epilepsy using subdural electrode arrays implanted as part of the standard presurgical evaluation process (Supplementary Table 3). Electrode array locations were thus based solely on the requirements of the clinical evaluation. Participants were recruited from three medical centers: Albany Medical Center (NY, USA), Geneva University Hospitals (Switzerland), and NYU Langone Medical Center (NY, USA). All participants gave informed consent, and the experiments reported here were approved by the respective ethical committees (Albany Medical College Institutional Review Board 18, Commission Cantonale d'Ethique de la Recherche, project number

2016-01856, and the Institutional Review Board at the New York University Langone Medical Center). No monetary compensation was given to the participants.

Note that full information on the approval of the study protocol must also be provided in the manuscript.
